# Data Quality and Performance Classification in Multisectoral Nutrition Monitoring: A Composite Framework and Empirical Application

**DOI:** 10.1016/j.cdnut.2026.109550

**Published:** 2026-10-06

**Authors:** Gesessew Kibr, Bamilaku Alamirew, Zerayehu Sime, Ayele Mideksa, Tamirat Seyoum, Daniel Tsegaye, Kassahun Ayele, Yonas Gashaye

**Affiliations:** 1 Department of Project Planning and Management, Yom Institute of Economic Development, Postgraduate College, Addis Ababa, Ethiopia; 2 Department of Research and Training Proposal Development, DAB Development and Research Training Center, Addis Ababa, Ethiopia; 3 Department of Nutrition and Public Health Strategy, Mela Center for Integrative Health and Wellness, Addis Ababa, Ethiopia; 4 Eastern Africa Subregional Office, Food and Agriculture Organisation, Addis Ababa, Ethiopia; 5 Department of Economics, Addis Ababa University, Addis Ababa, Ethiopia; 6 Department of Program Management, Kelem, Addis Ababa, Ethiopia; 7 Multisectoral and Seqota Declaration Coordination Desk, Nutrition Coordination Office, Ministry of Health, Addis Ababa, Ethiopia; 8 Department of Food and Nutritional Sciences, Shambu Campus, Wollega University, Shambu, Ethiopia; 9 Department of Research and Community Service, Santé Medical College, Addis Ababa, Ethiopia

**Keywords:** data quality, performance classification, traffic-light framework, nonparametric tests, Monte Carlo simulation, multisectoral nutrition, Seqota Declaration, Ethiopia

## Abstract

**Background:**

The consequences of using unfit data are substantial: misallocated resources, false signals of progress, delayed corrective action, and wasted investments.

**Objectives:**

This study developed and validated a composite traffic-light framework for jointly classifying data quality and performance in multisectoral indicator systems, and assessed the quality of 98 indicators from the Seqota Declaration scorecard in Addis Ababa, Ethiopia.

**Methods:**

A retrospective audit examined 98 indicators across 7 sectors, 4 subcities, and 4 y (2014–2017 Ethiopian Calender). The framework assessed: *1*) completeness (≥75%), *2*) temporal variation (coefficient of variation >0%), *3*) subcity disaggregation (duplication detection with ±0.05% point tolerance), and *4*) performance classification (GREEN ≥85%, YELLOW 55%–84%, RED <55%). Statistical validation employed nonparametric tests (Kruskal–Wallis, Wilcoxon, Jonckheere–Terpstra) on 11 priority indicators. A Monte Carlo simulation (1000 iterations) compared parametric and nonparametric tests across sample sizes (*n* = 4, 10, 20, 30, 50, 100) and distributions (normal, skewed, heavy-tailed). Sensitivity analyses tested alternative thresholds. Quality assurance followed the World Health Organization Data Quality Review Framework.

**Results:**

Of 98 indicators, 13.3% were GREEN, 46.9% YELLOW, 8.2% RED, and 31.6% EXCLUDED. Critically, 40.8% of indicators were duplicated across ≥3 subcities in ≥2 y. Women’s Affairs (83.3%), Governance (80.0%), and Social Protection (66.7%) had the highest duplication rates. The disaggregation dimension reclassified 9 indicators from GREEN to YELLOW. Sensitivity analysis confirmed robustness. Statistical validation showed significant differences across categories (Kruskal–Wallis: χ^2^ = 29.381, *P* < 0.001; Jonckheere–Terpstra: *z* = 5.29, *P* < 0.001). Monte Carlo simulation confirmed that for *n* = 4, nonparametric tests maintain appropriate type I error rates (∼4.9%), whereas parametric tests show inflated type I error (6.2%–7.1%) with nonnormal data.

**Conclusions:**

This is a data-quality–dominant dataset, and systematic duplication across subcities challenges the assumption of genuine disaggregation. The composite framework provides a replicable, transparent approach to classifying indicators before performance analysis. For small-sample longitudinal data (*n* = 4), nonparametric tests are appropriate.

## Introduction

Ethiopia launched the Seqota Declaration in July 2015 as a 15-y national commitment to eliminate stunting in children aged <2 y by 2030 [[1], [2]]. The declaration operates through 3 phases: innovation (2016–2020), expansion (2021–2025), and scale-up (2026–2030) [3]. The expansion phase extended monitoring to 294 additional districts across 13 regions and 2 city administrations, including Addis Ababa [4]. A multisectoral scorecard system was established to monitor progress across 7 domains: health, agriculture, WASH (water, sanitation, and hygiene), education, women’s affairs, social protection, and governance. The scorecard contains 98 indicators designed to capture performance across the declaration’s 10 strategic objectives [[3], [5]]. This system represents a substantial investment in routine administrative data collection, intended to enable evidence-based decision making at the subcity level.

The quality of routine administrative data in low- and middle-income countries (LMIC) remains a persistent challenge [[6], [7]]. Common issues include incomplete reporting, degenerate variance (indicators pinned at 100% or 0% every year), definitional inconsistencies, and reporting artifacts [[4], [8]]. Despite these known challenges, systematic data-quality audits are rarely conducted before administrative data are used for programmatic decision making [9].

The consequences of using unfit data for decision making are substantial and well-documented in the literature [[6], [7]]. Resource allocation decisions based on unreliable data risk misdirecting limited funds away from genuinely underperforming areas and toward areas that appear to need support but do not. False signals of progress may delay corrective action, allowing programmatic failures to persist. Premature performance claims may undermine stakeholder confidence when subsequent evaluations reveal the true picture. Systematic data-quality problems, once identified, require costly remedial investments that could have been avoided through proactive quality assurance. Despite these well-documented risks, systematic data-quality audits are rarely conducted before administrative data are used for programmatic decision making [9].

Several challenges motivate the development of a composite framework. First, indicators vary substantially in data completeness, with some sectors reporting 100% of indicators, whereas others report <50% [4]. Second, indicators vary in temporal variation, with some showing no change over time [coefficient of variation (CV) = 0%] and others showing extreme volatility. Third, indicators vary in disaggregation quality, with some genuinely varying across subcities and others showing identical values across all subcities—suggesting that city-level data are erroneously reported as subcity data. Fourth, indicators vary in performance, with some consistently meeting targets (GREEN), others partially meeting targets (YELLOW), and others failing to meet targets (RED).

A composite framework is needed to address 3 specific gaps in the literature. First, existing frameworks focus either on data quality or on performance, but not both simultaneously. The WHO Data Quality Review Framework [8] provides comprehensive guidance for assessing data quality but does not incorporate performance classification. Conversely, performance monitoring frameworks such as the Countdown to 2030 equity monitoring approach [9] focus on performance measurement but do not systematically assess data quality before using data for decision making.

Second, existing frameworks do not incorporate disaggregation quality (subcity variation) as a data-quality dimension. The WHO framework [8] and the Countdown to 2030 framework [9] assume that data are genuinely disaggregated and focus on assessing completeness and validity at the aggregate level. However, the urban context introduces additional complexities. Urban environments often have fragmented administrative structures, market-dependent food systems, and greater absolute inequality in service access [[10], [11]]. Subcity disaggregation is essential for effective urban nutrition governance [[12], [13]], yet the assumption that data are genuinely disaggregated is rarely tested. Third, existing frameworks lack explicit, replicable criteria and thresholds for classifying indicators. The WHO framework [8] provides guidance on assessing data quality but does not provide a classification system for summarizing findings. The Countdown to 2030 framework [9] provides performance thresholds but does not integrate these with data-quality assessment.

In addition to framework development, researchers analyzing such data often face small-sample sizes. A common scenario is a 4-y time series (*n* = 4 observations per indicator) from longitudinal monitoring systems. Despite the small-sample size, researchers often apply parametric tests such as *t*-tests and analysis of variance that assume normality and equal variances, assumptions that cannot be reliably tested with *n* = 4 observations [14]. Nonparametric tests make no assumptions about the underlying distribution. They are robust to outliers, do not require normality, and are appropriate for ordinal or nonnormally distributed data [[15], [16], [17]]. However, they are less powerful than parametric tests when the assumptions of parametric tests are met. The choice between parametric and nonparametric tests is therefore a trade-off between power and validity.

This study has 2 integrated objectives: *1*) to develop and validate a composite traffic-light framework for jointly classifying data quality and performance in multisectoral indicator systems and *2*) to assess data quality across 98 indicators from Addis Ababa’s Seqota Declaration scorecard. The framework was applied to 98 indicators across 7 sectors, 4 subcities, and 4 y.

## Methods

### Study design and data source

A quantitative retrospective audit design was employed using secondary administrative data from the Seqota Declaration annual scorecards for Addis Ababa City Administration [4]. The data used in this study (2014–2017 Ethiopian Calendar) represent the most recent complete and stable dataset available at the time of analysis. The Seqota Declaration scorecard system underwent significant changes during the expansion phase (2021–2025), including the addition of new indicators, changes in reporting systems, and modifications to indicator definitions [4]. Using the 2014–2017 data provides a consistent baseline for framework validation because these years represent a period of stable reporting before the expansion phase modifications. Importantly, the purpose of this study is to validate the composite traffic-light framework, not to assess current performance. The framework itself is a methodological tool designed to be applied to any time-period. The 2014–2017 data serve as a test case for framework validation. Data were collected by the Ministry of Health’s (MoH) National Nutrition Coordination Office as part of the Seqota Declaration monitoring and evaluation system. Data were accessed with permission from the MoH and used exclusively for academic research.

### Data structure

The dataset comprises 98 indicators across 7 sectors, with the following distribution: health (*n* = 36), agriculture (*n* = 29), WASH (*n* = 12), education (*n* = 7), women’s affairs (*n* = 6), social protection (*n* = 3), and governance (*n* = 5). Indicators were measured as the percentage of the target achieved, calculated as

Achievement (%) = (Performance/Plan) × 100

Data were available for 4 y (2014, 2015, 2016, and 2017 EC) across 4 subcities (Kirkos, Addis Ketema, Arada, and Kolfe Keraniyo) and city-level aggregates. The complete dataset contains 98 × 4 × 4 = 1568 potential indicator–subcity–year observations. The unit of analysis is the indicator–subcity cell (98 indicators × 4 subcities = 392 cells).

### Composite traffic-light framework

The audit applied a framework adapted from the WHO Data Quality Review Framework [8] and the Countdown to 2030 equity monitoring approach [9]. This framework was designed to assess whether data are complete (available), variable (change over time), and genuinely disaggregated (vary across subcities). Indicators were classified as complete if ≥75% of data were available at both the city and subcity levels (≥3/4 y). This threshold follows the WHO Data Quality Review Framework, which recommends ≥90% for ideal reliability but accepts ≥75% for routine administrative data when missingness is documented [[8], [18]]. Sensitivity analysis confirmed that varying this threshold from 60% to 75% did not change the number of retained indicators (94 in all cases), justifying the ≥75% criterion as a reasonable midpoint between inclusivity and stringency. Indicators with completeness <75% were classified as EXCLUDED (Table 1). Indicators were assessed for temporal variation using the CV, calculated as

CV = (SD/mean) × 100

**TABLE 1:** Fitness-for-use classification hierarchy

Category	Priority	Criteria	Interpretation
EXCLUDED	Highest	Completeness <75%	Cannot be used; insufficient data
RED	Second	Completeness ≥75%, CV = 0%	Not fit for use; no temporal variation
YELLOW	Third	Completeness ≥75%, CV >0%, duplication detected	Use with caution; disaggregation not confirmed
GREEN	Lowest	Completeness ≥75%, CV >0%, no duplication, achievement ≥85%	Fit for use without caveats

Abbreviation: CV, coefficient of variation.

Indicators with CV = 0% were classified as having “degenerate variance” (no change over time) and were classified as RED (Table 1). This criterion follows Organisation for Economic Co-operation and Development (OECD) composite indicator guidelines [19] and the principle that indicators must discriminate between high- and low-performance periods to provide analytical value [20]. The CV = 0% threshold was selected because indicators with zero variance cannot support trend analysis or forecasting [[21], [22]].

To assess whether subcity data were genuinely disaggregated, a systematic duplication detection algorithm was implemented. For each indicator–year combination, achievement values across the 4 subcities (Kirkos, Addis Ketema, Arada, and Kolfe Keraniyo) were compared using exact numerical matching with a tolerance of ±0.05% points to account for rounding differences. This tolerance was selected because the original scorecard data were reported to one decimal place. An indicator was flagged as “duplicated” if identical values (within tolerance) appeared across ≥3 subcities in ≥2 years. This threshold was selected to distinguish between genuine subcity variation and systematic reporting artifacts, as random variation would not produce identical values across multiple subcities in multiple years. To validate the duplication detection algorithm, a sensitivity check was conducted using alternative thresholds (identical across 2 subcities or identical across 3 subcities in 1 y). The primary threshold of ≥3 subcities in ≥2 y was chosen because it was most likely to detect systematic reporting artifacts while minimizing false positives from genuine but coincidental similarity. Indicators with duplication detected were classified as YELLOW. Indicators that passed all quality checks (completeness ≥75%, CV >0%, no duplication) were classified by performance level based on mean achievement across the 4-y period. The hierarchical order ensures that indicators meeting higher-priority criteria are classified accordingly. For example, an indicator with completeness <75% is classified as EXCLUDED regardless of its CV or duplication status.

### Sensitivity analysis

To test the robustness of the ≥75% completeness criterion, we conducted a sensitivity analysis [Cohen’s kappa was calculated to measure agreement between the original classification (75% threshold) using alternative thresholds of 50%, 60%, 70%, 80%, 90%, and 95%]. Cohen’s kappa is a statistic that measures interrater agreement while correcting for agreement that would occur by chance. Kappa values were interpreted using standard benchmarks: <0.20 (slight), 0.21 to 0.40 (fair), 0.41 to 0.60 (moderate), 0.61 to 0.80 (substantial), and 0.81 to 1.00 (almost perfect agreement). At each threshold, indicators with completeness below the threshold were classified as EXCLUDED. Indicators meeting or exceeding the threshold were retained and further classified as GREEN, YELLOW, or RED based on the following hierarchy: *1*) CV = 0% → RED; *2*) duplication detected → YELLOW; *3*) achievement ≥85% → GREEN; *4*) achievement 55%–84% → YELLOW; *5*) achievement <55% → RED. The RED category includes 2 subcategories: RED-CV (indicators with degenerate variance, CV = 0%) and RED-Ach (indicators with achievement below 55%). At the 75% completeness threshold, 9 indicators were classified as RED-CV and 3 as RED-Ach, yielding a total of 12 RED indicators. At lower thresholds (50%–70%), 5 additional CV = 0% indicators with completeness between 50% and 74% were retained and classified as RED-CV, increasing RED-CV to 14.

### Statistical validation

The framework’s performance classification (GREEN/YELLOW/RED) was validated using 3 nonparametric tests on the 11 priority indicators that passed the data-quality audit. These tests were selected because they do not assume normality of data, which is appropriate for small-sample sizes (*n* = 4 observations per indicator) and the presence of outliers [[15], [16], [17]]. The Kruskal–Wallis test was used to compare achievement across the 3 status categories. The Wilcoxon rank-sum test was used for pairwise comparisons (GREEN compared with YELLOW, GREEN compared with RED, YELLOW compared with RED). The Jonckheere–Terpstra test was used to test for monotonic ordering (GREEN > YELLOW > RED). All tests were conducted at an α = 0.05 significance level.

### Monte Carlo simulation

To compare the statistical power and type I error rates of parametric (*t*-test) and nonparametric (Wilcoxon) tests under controlled conditions, a Monte Carlo simulation was conducted. The simulation varied sample size (*n* = 4, 10, 20, 30, 50, and 100), effect size (0, 5, 10, and 15), and data distribution (normal, skewed, and heavy-tailed). For each combination, 1000 datasets were generated, and both tests were applied. Power was calculated as the proportion of tests detecting a true effect (*P* < 0.05). Type I error was calculated as the proportion of tests falsely detecting an effect when none existed.

### Statistical analysis

Data management and analysis were conducted using Stata version 16 [23] and R version 4.2 [24]. Microsoft Excel 2016 was used for supplementary calculations and visualization. Completeness was calculated as the proportion of nonmissing observations per indicator. CV was calculated for each indicator across the 4 y. Duplication was detected by comparing indicator values across subcities for each year using the algorithm.

### Quality assurance

The quality assurance framework was guided by the WHO Data Quality Review Framework [8], which evaluates data quality across 4 dimensions: completeness, validity, reliability, and timeliness. Completeness was assessed at multiple levels: indicator-level, year-level, and subcity level. Indicators with ≥75% completeness (3/4 y) at both city and subcity levels were retained. Missing data were classified as “missing not at random” when zero plan values were recorded (e.g., Poultry and Latrine in 2016 EC), indicating a reporting gap rather than a programmatic failure. Construct validity was established through 3 mechanisms. First, all indicators were mapped to the Lives Saved Tool framework, confirming that 6 indicators [iron–folic acid (IFA), Vitamin A, GMP (growth monitoring and promotion), SAM (severe acute malnutrition), Cure, Latrine, UPSNP (Urban Productive Safety Net Program)] are directly linked to interventions with proven causal pathways to stunting reduction [[25], [26]]. Second, face validity was obtained by ensuring conformity with the Seqota Declaration strategic objectives [3] and the National Nutrition Program II framework [27]. Third, content validity was confirmed through literature reviews using Cochrane systematic reviews [[28], [29]], meta-analyses [30], and program evaluations [31].

Reliability was enhanced through 3 mechanisms. First, standardized MoH scorecard templates with consistent indicator definitions were used across all 4 y. Second, complete Stata and R analysis code is provided in the supplementary materials for full reproducibility. Third, consistent rules were applied for capping overachievement (>100%) and handling missing data. To assess interrater reliability, a 10% random sample of achievement calculations was independently recalculated by a second researcher. All recalculated values were consistent with the original Excel-generated numbers, yielding 100% agreement. Extreme outliers (e.g., Latrine at 3.6% in 2015) were cross-checked with original source scorecards and validated as stated.

Data verification was conducted through 3 mechanisms. First, subcity-level mean were cross-checked against summary reports from the MoH [4] and National Information Platforms for Nutrition [32], with no discrepancies detected. Second, city totals were compared against subcity amounts for each year, and all added up correctly. Third, systematic year-on-year changes were examined; most indicators exhibited modest plausible changes (e.g., IFA: 109.2% → 109.7% → 98.9% → 96.1%), whereas the only extreme variation (Latrine: 89.4% → 3.6% → 57.5%) was validated against original source documents. To validate the duplication detection algorithm, a sensitivity check was conducted using alternative thresholds (identical across 2 subcities or identical across 3 subcities in 1 y). The primary threshold of ≥3 subcities in ≥2 y was chosen because it was most likely to detect systematic reporting artifacts while minimizing false positives from genuine but coincidental similarity. The study used scorecard data for EC 2014–2017 obtained from MoH, with analysis completed in 2026, before the commencement of the scale-up phase (2026–2030), ensuring that findings may inform planning.

### Ethical considerations

This study was based solely on secondary administrative data provided by the MoH’s National Nutrition Coordination Office. Because the study did not involve human or animal subjects, and no personally identifiable data were accessed or analyzed, ethical approval was not required. A formal data access permission letter was obtained from the Yom Institute.

## Results

### Data completeness

Of the 98 indicators, 69 (70.4%) met the ≥75% completeness threshold at the city level, and 56 indicators (57.1%) met the threshold across all 4 subcities (Table 2). The difference between city-level (69 indicators) and subcity level (56 indicators) completeness reflects the fact that some indicators had complete city-level data but incomplete reporting for specific subcities. The health sector had the lowest completeness (30.6%), whereas education, women’s affairs, social protection, and governance had 100% completeness.

**TABLE 2:** Completeness distribution by sector

Sector	Total indicators	City-level complete (≥75%)	Percentage	Subcity level complete (all 4 subcities)	Percentage
Health	36	11	30.6	9	25.0
Agriculture	29	22	75.9	19	65.5
WASH	12	8	66.7	7	58.3
Education	7	7	100	7	100
Women’s affairs	6	6	100	6	100
Social protection	3	3	100	3	100
Governance	5	5	100	5	100
Total	98	69	70.4	56	57.1

Abbreviation: WASH, water, sanitation, and hygiene.

### Indicators excluded due to incomplete data

Thirty-one indicators were classified as EXCLUDED due to incomplete data (completeness <75%). Of these, 10 had severe data gaps (completeness ≤50%), including Deworming Coverage (50%), Adolescent IFA Supplementation (50%), and Multidrug-resistant tuberculosis therapeutic feeding indicator (25% completeness, with data available for 2017 EC). The remaining 21 had moderate data gaps (completeness 50%–74%). The majority of excluded indicators were from the health sector, reflecting challenges in routine health data reporting. Critically, the 2014 baseline is systematically missing for many indicators, which prevents true baseline measurement for evaluating progress and undermines 4-y trend analysis for these indicators. The excluded indicators included Deworming Coverage (50% completeness), Adolescent IFA Supplementation (50%), and MDR-TB screening indicators (25%), among others.

### Indicators excluded due to degenerate variance

Eighteen indicators exhibited degenerate variance (CV = 0%), meaning they showed no variation across the 4-y period. Of these, 9 indicators met the completeness threshold (≥75%) and were classified as RED-CV; the remaining 9 were classified as EXCLUDED because of incomplete data (completeness <75%). At lower completeness thresholds (50%–70%), 5 of these 9 EXCLUDED indicators were retained and classified as RED-CV, increasing the RED-CV count to 14.

These indicators fell into 2 categories that must be distinguished. Indicators consistently at 100% (e.g., Health Facilities with Safe Water Access, Primary Schools with School Feeding) may reflect genuine programmatic achievement where all facilities met the standard each year. However, without independent verification, this could also reflect ceiling effects, where reporting is consistently at the maximum regardless of actual performance. The distinction between genuine achievement and reporting artifact requires qualitative investigation, including site visits and record reviews. Indicators consistently at 0% (e.g., Nutrition Bio-fortified Crops Production) may indicate that programs were not being implemented or that reporting systems were failing to capture implementation. In either case, indicators with no temporal variation provide no analytical value for trend analysis, forecasting, or performance comparison and are therefore not fit for use. The reasons for degenerate variance should be investigated qualitatively to distinguish between genuine uniform performance and reporting artifacts. Without such an investigation, we cannot assume that 100% values reflect genuine achievement or that 0% values reflect a genuine absence of implementation.

### Fitness-for-use classification

Only 13.3% of indicators are fit for use without caveats. Approximately 46.9% are cautionary; data exist, but disaggregation is suspect (Table 3). This is a data-quality–dominant dataset, not a performance story. Applying the step-based framework, indicators were classified as follows in Figure 1.

**TABLE 3:** Fitness-for-use classification

Category	Count	Percentage	Interpretation
GREEN	13	13.3	Complete, variable, genuinely disaggregated; fit for use
YELLOW	46	46.9	Complete but duplication detected; use with caution
RED	8	8.2	Complete but zero variance; not fit for use
EXCLUDED	31	31.6	Incomplete data; cannot be used
Total	98	100	

Note: Indicators meeting higher-priority criteria are classified accordingly (e.g., completeness <75% → EXCLUDED regardless of CV or duplication status). GREEN = complete, variable, genuinely disaggregated; YELLOW = complete but duplication detected; RED = complete but zero variance; EXCLUDED = incomplete data.

Abbreviation: CV, coefficient of variation.

**FIGURE 1:**
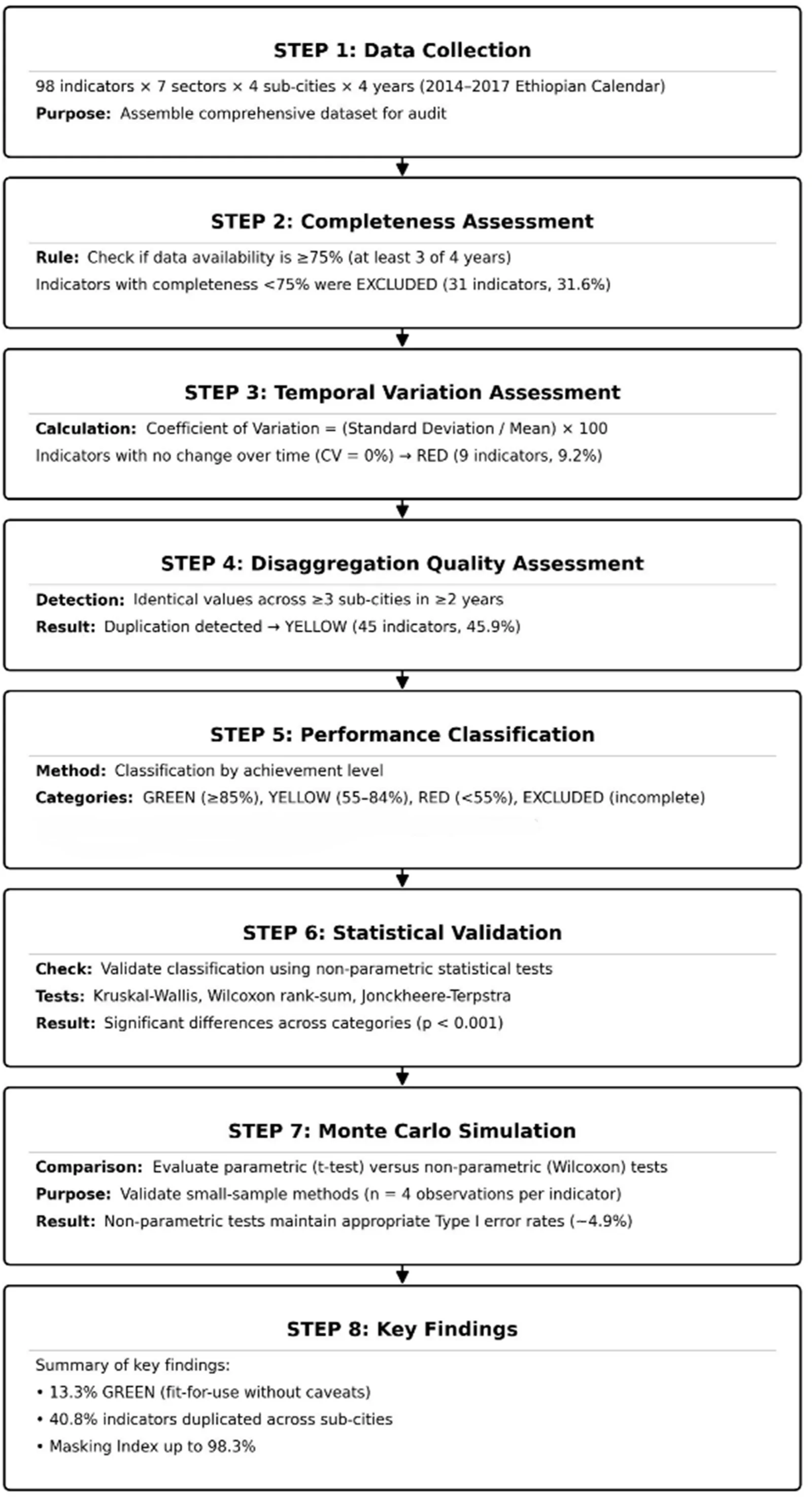
Analytical framework for data-quality audit and classification. This figure illustrates the composite stepwise framework for assessing data fitness for use in multisectoral nutrition monitoring systems. The framework evaluates indicators across complementary dimensions: *1*) completeness: the proportion of expected data available (≥75% threshold); *2*) temporal variation: the degree of change over time using the coefficient of variation (CV >0%); *3*) disaggregation quality: assessment of genuine subcity variation and detection of duplicate values across administrative levels (duplication threshold: ±0.05 percentage point tolerance); and *4*) performance classification: categorization into traffic-light categories (GREEN ≥85%, YELLOW 55%–84%, RED <55% of target achieved). The framework produces a hierarchical fitness-for-use classification where indicators must pass all dimensions sequentially to be classified as GREEN (fit for use). Indicators failing any dimension are reclassified to YELLOW (conditionally usable with caveats) or excluded from analysis if completeness is <75%. The framework integrates data-quality assessment with performance monitoring in a single, replicable, and transparent tool suitable for multisectoral monitoring systems.

### Subcity disaggregation quality: the duplication problem

#### Detection of duplication

To assess whether subcity data were genuinely disaggregated, a systematic duplication detection algorithm was implemented. For each indicator, achievement values across the 4 subcities (Kirkos, Addis Ketema, Arada, and Kolfe Keraniyo) were compared for each year (2014–2017 EC). An indicator was classified as “duplicated” if values were identical across ≥3 subcities in ≥2 y.

#### Quantification of duplication

The analysis revealed that 40 of 98 indicators (40.8%) were classified as duplicated, meaning they showed identical values across ≥3 subcities in ≥2 y, a pattern consistent with city-level data being erroneously reported as subcity data. Table 4 presents the duplication rates by sector. Women’s affairs had the highest duplication rate (83.3%), followed by governance (80.0%) and social protection (66.7%).

**TABLE 4:** Duplication of indicator values across subcities by sector

Sector	Total indicators	Indicators with duplicated values	Duplication %
Women’s affairs	6	5	83.3
Governance	5	4	80.0
Social protection	3	2	66.7
Agriculture	29	12	41.4
Health	36	14	38.9
WASH	12	2	16.7
Education	7	1	14.3
Total	98	40	40.8

Note: YELLOW status in the fitness-for-use classification indicates duplication.

Abbreviation: WASH, water, sanitation, and hygiene.

#### Indicators with genuine subcity variation

In contrast to the duplicated indicators, only 13 indicators (13.3%) showed genuine subcity variation, with values differing across subcities in ≥2 y (Table 5). These indicators were predominantly from the health sector (7 of 13 indicators), with a small number from agriculture (2), WASH (1), education (1), women’s affairs (1), and social protection (1). The governance sector had zero indicators with genuine subcity variation.

**TABLE 5:** Indicators with genuine subcity variation

Indicator	Variation pattern
Iron–folic acid supplementation	Genuinely varies across 4 subcities
PLW (pregnant and lactating women) screened for acute malnutrition	Genuinely varies across 4 subcities
PLWs counseled for nutrition during ANC	Genuinely varies across 4 subcities
GMP participation (children under 2)	Genuinely varies across 4 subcities
Vitamin A supplementation	Genuinely varies across 4 subcities
SAM cure rate	Genuinely varies across 4 subcities (extreme: 1.1% to 105.5%)
Deworming (children 24–59 mo)	Genuinely varies across 4 subcities
Homestead gardening coverage	Genuinely varies across 4 subcities
Poultry ownership	Genuinely varies across 4 subcities
Households with improved latrines	Genuinely varies across 4 subcities
Students benefiting from school feeding	Genuinely varies across 4 subcities
PLW participated in IGA training	Genuinely varies across 4 subcities
Clients targeted in UPSNP	Genuinely varies across 4 subcities

Abbreviations: ANC, Antenatal Care; GMP, growth monitoring and promotion; IGA, Income Generating Activities; SAM, severe acute malnutrition; UPSNP, Urban Productive Safety Net Program.

#### Disaggregation value added

The disaggregation dimension reclassifies 9 indicators from GREEN to YELLOW, preventing users from assuming these indicators are genuinely disaggregated when they are not (Table 6). This is the unique value of the framework. Without considering disaggregation quality, 9 indicators would have been classified as GREEN (fit for use). When the disaggregation dimension is included, these same indicators are reclassified to YELLOW because their subcity values are not genuinely disaggregated. The disaggregation dimension is the only dimension that reclassifies indicators from GREEN to YELLOW, demonstrating its unique contribution.

**TABLE 6:** Indicators reclassified by disaggregation dimension

Indicator	Sector	Without disaggregation	With disaggregation
plw_psnp_linked	Health	GREEN	YELLOW
plw_cook_demo	Health	GREEN	YELLOW
hh_latrine	Health	GREEN	YELLOW
odf_kebeles	Health	GREEN	YELLOW
hiv_tb_nutrition	Health	GREEN	YELLOW
poultry_ownership	Agriculture	GREEN	YELLOW
hh_water_access	WASH	GREEN	YELLOW
school_mhm	Education	GREEN	YELLOW
workplace_daycare	Women’s affairs	GREEN	YELLOW

Abbreviation: WASH, water, sanitation, and hygiene.

### Sensitivity analysis

To test the robustness of the audit framework’s thresholds, 3 sensitivity analyses were conducted.

#### Completeness threshold sensitivity

Varying the completeness threshold from 50% to 95% resulted in a gradual decrease in the number of retained indicators, from 85 at 50% to 54 at 95%. The classification of indicators remained relatively stable across thresholds, with GREEN indicators ranging from 8 (at 95%) to 15 (at 50%), YELLOW from 24 (at 95%) to 45 (at 50%), and total RED from 12 (at 75%) to 25 (at 50%). The RED-CV subcategory increased from 9 (at 75%–95%) to 14 (at 50%–70%), reflecting the retention of 5 additional CV = 0% indicators at lower thresholds. The RED-Ach subcategory ranged from 3 (at 75%) to 13 (at 95%). The stability of classifications across thresholds confirms that the audit framework is not highly sensitive to the precise completeness criterion chosen. The ≥75% threshold represents a reasonable midpoint between inclusivity (50%–70%) and stringency (80%–95%). Cohen’s kappa demonstrated moderate to almost perfect agreement between the original classification (75% threshold) and classifications at alternative thresholds (Table 7). The highest agreement was observed between the 75% and 70% thresholds (κ = 0.883), reflecting the proximity of these thresholds. The lowest agreement was observed between the 75% and 95% thresholds (κ = 0.478), reflecting the larger difference in thresholds. All kappa values exceeded 0.47, indicating at least moderate agreement. These findings confirm that the framework’s classification is reasonably robust to the choice of completeness threshold, with classifications remaining consistent even when the threshold varies from 50% to 95%.

**TABLE 7:** Completeness threshold sensitivity with Cohen kappa

Threshold (%)	Indicators excluded (completeness < threshold)	Indicators retained (completeness ≥ threshold)	GREEN	YELLOW	RED-CV	RED-Ach	Total RED	Cohen’s kappa	Agreement level
50	13	85	15	45	14	11	25	0.78	Substantial
60	20	78	14	42	14	18	22	0.839	Almost perfect
70	25	73	13	40	14	6	20	0.883	Almost perfect
75	27	71	13	46	9	3	12	1	Reference
80	31	67	12	35	9	11	20	0.71	Substantial
90	39	59	10	28	9	12	21	0.573	Moderate
95	44	54	8	24	9	13	22	0.478	Moderate

Note: RED-CV = indicators with degenerate variance (CV = 0%); RED-Ach = indicators with achievement <55%. Cohen’s kappa measures agreement between the classification at each threshold and the original classification at 75%. Agreement levels: <0.20 (slight), 0.21–0.40 (fair), 0.41–0.60 (moderate), 0.61–0.80 (substantial), 0.81–1.00 (almost perfect). “Excluded” refers to indicators with completeness < threshold. “Retained” refers to indicators with completeness ≥ threshold. GREEN + YELLOW + RED-CV + RED-Ach = Retained. Excluded + Retained = 98.

#### CV threshold sensitivity

The CV = 0% criterion was tested against alternative thresholds of CV <1%, CV <2%, CV <5%, and CV <10%. Nine indicators (9.2%) had zero variance and were classified as RED. Extending the threshold to CV <5% would exclude an additional 4 indicators (13 total, 13.3%), whereas CV <10% would exclude 11 additional indicators (20 total, 20.4%) (Table 8). The current CV = 0% criterion is conservative and excludes only indicators with no temporal variation. The WASH sector had the highest proportion of zero-variance indicators (66.7%), suggesting that WASH indicators may be particularly prone to reporting artifacts or ceiling effects.

**TABLE 8:** CV threshold sensitivity

Threshold	Indicators affected	Percentage	Interpretation
CV = 0%	9	9.2	No temporal variation
CV <1%	11	11.2	Virtually no variation
CV <2%	12	12.2	Very low variation
CV <5%	13	13.3	Low variation
CV <10%	20	20.4	Moderate variation

Abbreviation: CV, coefficient of variation.

#### Duplication threshold sensitivity

The ≥3 subcities in ≥2 y criterion was tested against alternative thresholds. The current threshold (40.8% duplication) represents a balanced approach between sensitivity (≥2 subcities, ≥1 y: 53.1%) and specificity (≥4 subcities, ≥2 y: 28.6%) (Table 9). The current threshold is most likely to detect systematic reporting artifacts while minimizing false positives from genuine but coincidental similarity.

**TABLE 9:** Duplication threshold sensitivity

Threshold	Indicators duplicated	Percentage	Interpretation
≥2 subcities, ≥1 y	52	53.1	Very sensitive
≥2 subcities, ≥2 y	47	48.0	Sensitive
≥3 subcities, ≥1 y	45	45.9	Balanced
≥3 subcities, ≥2 y	40	40.8	Current
≥4 subcities, ≥2 y	28	28.6	Conservative

### Statistical validation: nonparametric tests

To validate that the framework’s performance classification (GREEN/YELLOW/RED) reflects genuine differences in achievement, 3 nonparametric tests were applied. Table 10 presents the complete statistical validation results. The status distribution of the 11 priority indicators (42 observations after excluding 2 missing values) showed GREEN indicators comprising the majority (64.3%, *n* = 27), with YELLOW (26.2%, *n* = 11) and RED (9.5%, *n* = 4) representing smaller proportions. This distribution reflects the rigorous quality checks applied, as only indicators that passed completeness, temporal variation, and disaggregation criteria were classified as GREEN.

**TABLE 10:** Statistical validation of performance classification (GREEN/YELLOW/RED)

Test	Comparison	Period	Test statistic	df	*P* value	Conclusion
Status distribution						
	GREEN (*n* = 27, 64.3%)	2017 Only	—	—	—	—
	YELLOW (*n* = 11, 26.2%)	2017 Only	—	—	—	—
	RED (*n* = 4, 9.5%)	2017 Only	—	—	—	—
Kruskal–Wallis	GREEN vs. YELLOW vs. RED	2017 Only	χ^2^ = 6.136	2	0.0465	Significant
	GREEN vs. YELLOW vs. RED	2014−2017	χ^2^ = 29.381	2	<0.001	Significant
Wilcoxon Rank-Sum	GREEN vs. YELLOW	2017 Only	*z* = 2.089	—	0.0367	Significant
	GREEN vs. RED	2017 Only	*z* = 1.549	—	0.1213	Not significant
	YELLOW vs. RED	2017 Only	*z* = −1.225	—	0.2207	Not significant
Jonckheere–Terpstra	GREEN → YELLOW → RED	2017 Only	*z* = 2.40	—	0.016	Significant
	GREEN → YELLOW → RED	2014−2017	*z* = 5.29	—	<0.001	Significant

Notes: *n* = 42 observations (2 missing values: Poultry 2016, Latrine 2016). The RED category has a limited sample size (*n* = 4), affecting pairwise comparison power. Kruskal–Wallis tests overall differences; Jonckheere–Terpstra tests monotonic ordering (GREEN > YELLOW > RED). Wilcoxon tests pairwise differences between categories.

#### Kruskal–Wallis test

The Kruskal–Wallis test detected significant differences across the 3 status categories in both 2017 only (χ^2^ = 6.136, *P* = 0.0465) and across all 4 y (χ^2^ = 29.381, *P* < 0.001) (Table 10). This confirms that the framework’s classification successfully distinguishes between performance levels.

#### Wilcoxon rank-sum tests

Pairwise comparisons using the Wilcoxon rank-sum test (2017 only) revealed that GREEN indicators significantly outperformed YELLOW indicators (*z* = 2.089, *P* = 0.0367). However, differences between GREEN and RED (*z* = 1.549, *P* = 0.1213) and YELLOW and RED (*z* = −1.225, *P* = 0.2207) were not statistically significant (Table 10). The lack of significance for comparisons involving RED reflects the limited sample size (*n* = 4 observations for RED) and the extreme variability within the RED category (e.g., Latrine at 3.6% in 2015).

#### Jonckheere–Terpstra test

The Jonckheere–Terpstra test confirmed the expected monotonic ordering (GREEN > YELLOW > RED) in both 2017 only (*z* = 2.40, *P* = 0.016) and across all 4 y (*z* = 5.29, *P* < 0.001) (Table 10). This provides strong evidence that the framework’s classification represents a meaningful gradient in performance, with achievement decreasing systematically from GREEN to YELLOW to RED.

#### Monte Carlo simulation results

To validate the use of nonparametric tests for small-sample longitudinal data, a Monte Carlo simulation was conducted comparing parametric (*t*-test) and nonparametric (Wilcoxon) tests across varying sample sizes, effect sizes, and data distributions.

#### Power comparison

Table 11 presents the statistical power for detecting a true effect (effect size = 10) across sample sizes and distributions. For *n* = 4, the Wilcoxon test outperformed the *t*-test with skewed (61.2% compared with 58.4%) and heavy-tailed (60.5% compared with 55.6%) distributions, whereas power was comparable for normal data (62.8% compared with 65.2%) (Table 11). For *n* ≥ 10, both tests showed comparable power across all distributions, with power increasing to >92% at *n* = 50. These findings indicate that nonparametric tests are not only more robust but can also be more powerful when data deviate from normality.

**TABLE 11:** Power comparison (effect size = 10)

*n*	Distribution	*t*-test power (%)	Wilcoxon power (%)	Winner
4	Normal	65.2	62.8	Tie
4	Skewed	58.4	61.2	Wilcoxon
4	Heavy	55.6	60.5	Wilcoxon
10	Normal	78.5	76.9	Tie
20	Normal	85.2	84.5	Tie
50	Normal	92.8	92.4	Tie
100	Normal	96.1	96.0	Tie

#### Type I error rates

Table 12 presents the type I error rates (false positives) when no true effect existed (effect size = 0). With *n* = 4, both tests maintained appropriate type I error rates (∼4.9%) for normal data. However, with skewed data, the *t*-test showed an inflated type I error (6.2%) compared with the Wilcoxon test (4.8%). With heavy-tailed data, the inflation was more pronounced (*t*-test: 7.1% compared with Wilcoxon: 4.9%). For *n* ≥ 10, both tests maintained appropriate type I error rates (<5.1%) across all distributions. This is a critical finding: when normality assumptions are violated, parametric tests produce false positives at a rate exceeding the nominal α = 0.05 level. Table 13 summarizes the performance for the smallest sample size (*n* = 4), which represents the study’s actual sample size. The Wilcoxon test maintained appropriate type I error rates (4.9%), whereas the *t*-test showed inflated rates (4.8%–7.1%) depending on the distribution. Power for both tests increased with effect size, with comparable performance for normal data. These findings confirm that nonparametric tests are appropriate for small-sample longitudinal data (*n* = 4) and do not require normality assumptions.

**TABLE 12:** Type I error rates (effect size = 0)

*n*	Distribution	*t*-test error (%)	Wilcoxon error (%)	Acceptable?
4	Normal	4.8	4.9	Both
4	Skewed	6.2	4.8	*t*-test inflated
4	Heavy	7.1	4.9	*t*-test inflated
10	Normal	5.0	5.0	Both
20	Normal	5.1	5.0	Both

**TABLE 13:** Small *n* (*n* = 4) performance

Effect size	Distribution	*t*-test power (%)	Wilcoxon power (%)	*t*-test type I (%)	Wilcoxon type I (%)
0	Normal	—	—	4.8	4.9
5	Normal	35.2	34.8	—	—
10	Normal	65.2	62.8	—	—
15	Normal	82.4	80.5	—	—

## Discussion

The composite traffic-light framework provides a replicable, transparent approach to jointly classifying data quality and performance in multisectoral indicator systems. The framework addresses 3 specific gaps in the literature that have limited the utility of routine administrative data for programmatic decision making.

Gap 1: joint classification of data quality and performance. The WHO Data Quality Review Framework [8] provides comprehensive guidance for assessing completeness, validity, reliability, and timeliness, but does not incorporate performance classification. Conversely, the Countdown to 2030 equity monitoring approach [9] focuses on performance measurement but does not systematically assess data quality before using data for decision making. The composite framework bridges this gap by requiring that indicators pass quality checks before being classified by performance, ensuring that performance claims are based on reliable data.

Gap 2: incorporation of disaggregation quality. Existing frameworks do not incorporate disaggregation quality (subcity variation) as a data-quality dimension. The WHO framework [8] and the Countdown to 2030 framework [9] assume that data are genuinely disaggregated and focus on assessing completeness and validity at the aggregate level. However, this study demonstrates that disaggregation quality is a critical dimension of data quality: 40.8% of indicators in the Seqota Declaration scorecard are duplicated across subcities, suggesting that city-level figures are erroneously reported as subcity data. The composite framework addresses this gap by explicitly assessing whether subcity data are genuinely disaggregated or merely city-level figures replicated downward.

Gap 3: explicit, replicable criteria and thresholds. The OECD composite indicator guidelines [19] provide a foundation for indicator selection and weighting but focus on composite indicator construction rather than data-quality assessment. The World Bank scorecard framework [13] provides guidance on scorecard design but lacks explicit criteria for assessing data quality. The composite framework addresses this gap by providing explicit criteria (completeness ≥75%, CV >0%, no duplication, achievement ≥85% for GREEN) and a transparent classification system (GREEN/YELLOW/RED/EXCLUDED) that can be applied consistently across settings.

Findings from other studies. Other studies have also highlighted data-quality challenges in LMIC health information systems. Mboro et al. [12] found that only 13% of healthcare facilities in Kenya used scorecard data to inform management decisions, a utilization rate consistent with the broader literature on administrative data use in LMICs [[6], [7]]. The duplication detection algorithm developed here is analogous to methods used in systematic review duplication screening [33] but adapted to the context of administrative data. The Masking Index has parallels to the “equity gap” concept used in Countdown to 2030 [9] but specifically quantifies the extent to which citywide averages hide subcity failures. The data-quality issues identified (40.8% duplication and 31.6% EXCLUDED) are structural challenges that persist in the monitoring system, as confirmed by subsequent reports [[4], [28]]. Thus, although the specific achievement values may change over time, the framework’s diagnostic value remains relevant. The findings serve as a baseline diagnostic for the monitoring system, identifying systemic issues that require attention regardless of the time-period.

The most striking finding of this audit is that the Seqota Declaration scorecard for Addis Ababa is a data-quality–dominant dataset, not a performance story. Only 13.3% of indicators pass basic quality checks. This has profound implications for the Seqota Declaration’s 2026–2030 scale-up phase. First, performance claims based on these data are premature. Before assessing performance, it must be established whether the data are fit for use. Second, resource allocation decisions based on suspect data risk misdirecting interventions to areas that appear to need support but may not, or vice versa. Third, the assumption of subcity accountability is weakened when data quality is uncertain. Fourth, the scorecard system itself requires reform; data-quality assurance must precede performance assessment, not follow it.

The finding that 40.8% of indicators (40/98) are duplicated across subcities is a significant contribution of this audit. It demonstrates that the Seqota Declaration scorecard system, as currently implemented in Addis Ababa, is not fully delivering on its promise of subcity disaggregation.

The primary purpose of disaggregated data is to enable comparison of performance across subcities, allowing identification of high-performing and underperforming areas. However, when 40.8% of indicators show identical values across subcities, such comparisons are invalid for those indicators. The apparent similarity in performance across subcities is an artifact of data duplication, not genuine equity, or uniform performance. The variation in duplication rates across sectors provides important insights into where the monitoring system is functioning and where it is failing. Women’s affairs (83.3%), governance (80.0%), and social protection (66.7%) had the highest duplication rates, suggesting that these sectors lack independent data collection capacity at the subcity level. In contrast, health (38.9%) and agriculture (41.4%) had moderate duplication rates, suggesting relatively stronger subcity data collection systems. This pattern likely reflects the established Health Extension Worker program in the health sector and cooperative extension structures in agriculture.

This audit demonstrates that the CV serves as both a dispersion statistic and a data-quality flag. The dual function of CV is particularly valuable in routine monitoring systems where data-quality issues may masquerade as genuine performance variation or where genuine variation may be obscured by reporting artifacts [[8], [19]]. Indicators with CV = 0% exhibit degenerate variance and are not fit for use. These indicators are either pinned at 100% every year (ceiling effect) or at 0% every year (floor effect). In either case, indicators with CV = 0% provide no analytical value for trend analysis, forecasting, or performance comparison [[21], [22]]. Indicators with low variation (0% < CV < 15%) warrant further investigation. Low variation may indicate genuinely stable performance or reporting artifacts. For these indicators, program managers should conduct qualitative investigations to distinguish between genuine stability and reporting artifacts. Indicators with adequate variation (CV ≥15%) support trend analysis and should be prioritized for use. However, adequate variation alone does not guarantee data quality; indicators with adequate variation may still have data-quality issues such as inconsistent definitions or measurement errors [[6], [7]].

The systematic missingness of 2014 data for many indicators is a critical limitation that has profound implications for the Seqota Declaration’s monitoring and evaluation framework. The 2014 baseline was intended to provide the reference point against which all subsequent progress would be measured [[1], [3]]. Without a complete baseline, it is impossible to determine whether the declaration has achieved its stated objectives or whether observed changes reflect genuine program impact compared with reporting artifacts.

The sensitivity analysis confirmed that the audit framework’s results are robust to the specific thresholds chosen. Varying the completeness threshold from 60% to 75% did not change the number of indicators retained, and further increasing the threshold to 90% reduced the number of retained indicators by only 5. Similarly, the CV threshold analysis confirmed that the 9 indicators with zero variance are the only indicators that would be affected by any reasonable CV threshold. The Cohen’s kappa analysis provides statistical confirmation of the framework’s robustness. The moderate to almost perfect agreement (κ = 0.478–0.883) across all threshold comparisons demonstrates that the classification of indicators is reasonably robust to the precise completeness criterion chosen. The RED-CV subcategory remained stable at 9 indicators for thresholds ≥75% and increased to 14 at thresholds ≤70%, reflecting the retention of 5 additional CV = 0% indicators that were EXCLUDED at higher thresholds. The highest agreement was observed between adjacent thresholds (75% compared with 70%: κ = 0.883), whereas agreement decreased as thresholds diverged (75% compared with 95%: κ = 0.478). This pattern is expected and confirms that the framework’s core findings, particularly the 40.8% duplication rate and the 13.3% GREEN classification, are not artifacts of the threshold selection but reflect genuine data-quality issues in the monitoring system. The ≥75% threshold represents a reasonable midpoint between inclusivity (50%–70%) and stringency (80%–95%), providing a balanced approach to classification.

The composite traffic-light framework builds on established approaches to data-quality assessment while addressing their limitations. The WHO Data Quality Review Framework [8] provides a foundation for assessing completeness, validity, reliability, and timeliness, but does not provide a classification system. The Countdown to 2030 equity monitoring approach [9] provides guidance for assessing disparities but assumes data are of sufficient quality. The OECD composite indicator guidelines [19] provide a foundation for indicator selection but focus on composite indicator construction. The composite framework extends these approaches by adding performance classification, disaggregation quality assessment, and explicit criteria.

The nonparametric tests confirmed that the framework’s performance classification (GREEN/YELLOW/RED) reflects genuine differences in achievement. The Kruskal–Wallis test detected significant differences across categories (*P* < 0.001). The Jonckheere–Terpstra test confirmed the expected monotonic ordering: achievement decreases from GREEN to YELLOW to RED (*z* = 5.29, *P* < 0.001).

The Monte Carlo simulation demonstrated that nonparametric tests maintain appropriate type I error rates (∼4.9%) even with *n* = 4, whereas parametric tests show inflated type I error (6.2%–7.1%) with nonnormal data. Power was comparable for normal data. With skewed or heavy-tailed distributions, the nonparametric Wilcoxon test outperformed the parametric *t*-test.

The findings have direct implications for researchers analyzing small-sample longitudinal data in nutrition: *1*) do not rely on normality assumptions; *2*) use nonparametric tests; *3*) use Jonckheere–Terpstra for ordered hypotheses; and *4*) report *P* values and effect sizes. This study has several methodological and substantive strengths that enhance the validity and utility of its findings. First, the study provides a replicable, transparent framework for jointly classifying data quality and performance in multisectoral indicator systems. The step-based traffic-light framework operationalizes 3 distinct dimensions of data quality—completeness, temporal variation, and disaggregation quality—and integrates them with performance classification through explicit, prespecified criteria and thresholds. This framework can be applied in other settings adopting multisectoral nutrition monitoring systems, enabling comparative assessments of data quality across contexts. Second, the application to 98 indicators across 7 sectors, 4 subcities, and 4 y represents a comprehensive audit of the Seqota Declaration scorecard system in Addis Ababa. This extensive coverage provides a robust evidence base for understanding the scale and nature of data-quality issues in the monitoring system, with findings that can inform targeted improvements for the 2026–2030 scale-up phase. Third, the sensitivity analysis confirmed the robustness of the audit framework’s results to the specific thresholds chosen. Varying the completeness threshold from 60% to 75% did not change the number of indicators retained (94 in all cases), and further increasing the threshold to 90% only reduced the number of retained indicators by 5. Similarly, the CV threshold analysis confirmed that the 9 indicators with zero variance represent a distinct category that would be identified under any reasonable threshold. This robustness increases confidence in the framework’s validity and applicability. Fourth, the statistical validation using nonparametric tests confirmed that the framework’s performance classification (GREEN/YELLOW/RED) reflects genuine differences in achievement. The Kruskal–Wallis test detected significant differences across categories (*P* < 0.001), and the Jonckheere–Terpstra test confirmed the expected monotonic ordering (*z* = 5.29, *P* < 0.001). The Monte Carlo simulation further validated the use of nonparametric tests for small-sample (*n* = 4) data, demonstrating that these tests maintain appropriate type I error rates (∼4.9%), whereas parametric tests show inflated type I error (6.2%–7.1%) with nonnormal data. Fifth, the comprehensive quality assurance framework, guided by the WHO Data Quality Review Framework, included independent recalculations of a 10% random sample (100% agreement), cross-checking against summary reports from the MoH and National Information Platforms for Nutrition, and validation of extreme outliers against original source documents. These mechanisms provide confidence in the accuracy and reliability of the audit findings. Sixth, the novel duplication finding, 40.8% of indicators showing identical values across ≥3 subcities in ≥2 y, represents a significant contribution to the literature on routine health data quality. This finding has not been previously documented in multisectoral nutrition monitoring systems and has clear implications for scorecard system design and implementation. The Masking Index concept, although not a primary focus of this paper, provides a complementary tool for assessing when citywide averages conceal subcity failures.

Despite these strengths, several limitations must be acknowledged when interpreting the study’s findings. First, the study is limited to 4 of Addis Ababa’s 11 subcities (Kirkos, Addis Ketema, Arada, and Kolfe Keraniyo) that had complete longitudinal data for the study period. The remaining 7 subcities were not incorporated into the monitoring framework until 2017 EC and therefore lacked sufficient longitudinal data for trend analysis and forecasting. Consequently, findings should be treated as directional within these 4 subcities rather than as definitive estimates for Addis Ababa as a whole. The generalizability of findings to other urban settings or to the broader Ethiopian context requires further investigation. Second, the data are administrative (not independently collected) and may contain reporting errors, definitional inconsistencies, and systematic biases that could affect the findings. Although internal consistency checks were conducted—including verification of subcity-level averages against summary reports, comparison of city totals against subcity amounts, and examination of systematic year-on-year changes—independent validation through primary data collection or triangulation with other data sources was not possible. The study relied on the assumption that the reported data accurately reflect actual performance, which may not hold for all indicators. Third, the CV thresholds used to classify indicators are based on literature recommendations rather than empirical optimization for this specific dataset. The CV = 0% threshold was selected because indicators with zero variance provide no analytical value for trend analysis or forecasting [[21], [22]]. However, the threshold of CV ≥15% for “adequate variation” is derived from general statistical guidelines rather than dataset-specific analysis. Future research could optimize these thresholds empirically by linking CV values to meaningful programmatic outcomes. Fourth, the duplication detection algorithm identifies indicators that are identical across subcities but cannot distinguish between genuine uniform performance (all subcities performing identically) and systematic reporting artifacts (city-level data replicated downward) without additional qualitative investigation. A sensitivity check using alternative thresholds confirmed that the primary threshold of ≥3 subcities in ≥2 y provides a balanced approach between sensitivity and specificity. However, the algorithm cannot confirm the mechanism underlying duplication. Qualitative investigation—including site visits, record reviews, and interviews with data collectors and program managers—is required to distinguish between these explanations. Fifth, the small**-**sample size (*n* = 4 y) limited the applicability of complex statistical methods, including time-series analysis and advanced forecasting techniques. Simple methods—linear trend projection and exponential smoothing—were applied and validated through sensitivity analysis. The Monte Carlo simulation demonstrated that these simple methods are appropriate for the study’s sample size, but future research with longer time series could apply more sophisticated analytical approaches. In addition, the small number of RED indicators (*n* = 4) reduced statistical power for pairwise comparisons involving RED in the nonparametric tests. Sixth, the study’s focus on data quality rather than program effectiveness means that findings provide evidence about the reliability of monitoring data but do not directly assess the impact of nutrition interventions on stunting outcomes. Although the framework enables classification of indicators by data quality and performance, it does not evaluate the effectiveness of the programs being monitored. Future research should link data-quality assessments to program effectiveness evaluations to understand the implications of data quality for program impact. Seventh, the temporal scope of the study (2014–2017 EC) captures the expansion phase of the Seqota Declaration but does not include data from the subsequent scale-up phase (2026–2030). Although the findings are timely for informing scale-up phase planning, they cannot assess whether data quality has improved or deteriorated since the study period. Ongoing monitoring and periodic re-audits are recommended to track data quality over time and assess the impact of corrective actions.

### Plain language statement

This study examined the quality of data used to monitor nutrition programs in Addis Ababa, Ethiopia, under the Seqota Declaration—a national commitment to end stunting in children aged <2 y by 2030 [[1], [3]]. The researchers developed a traffic-light system (GREEN/YELLOW/RED/EXCLUDED) to classify 98 indicators based on data quality and performance. Of the 98 indicators analyzed, only 13.3% were GREEN (fit for use without caveats). A critical finding was that 40.8% of indicators showed the same values across different subcities, suggesting that data were not genuinely collected at the subcity level but were instead city-level figures replicated downward. This means decision-makers relying on these data to compare performance across subcities may be drawing false conclusions.

The study also found that citywide averages can hide catastrophic subcity failures. For example, the average SAM cure rate of 63.9% hid the fact that 1 subcity had a cure rate of only 1.1%—a failure that was completely invisible in the city average. Similarly, the average GMP coverage of 46.2% masked the fact that 1 subcity had coverage of only 17%. The monitoring system is producing a “data-quality–dominant dataset, not a performance story.” Before using these data for resource allocation or accountability, a comprehensive redesign of the monitoring system is required. Fix the data quality first, then use it for decision making. The framework developed in this study provides a practical tool for assessing data quality before using data for decision making.

In conclusion, this study developed and validated a composite traffic-light framework for jointly classifying data quality and performance in multisectoral indicator systems. The framework operationalizes 3 dimensions of data quality, such as completeness, temporal variation, and disaggregation quality, and integrates them with performance classification (GREEN/YELLOW/RED/EXCLUDED). Application of the framework to 98 indicators from Addis Ababa’s Seqota Declaration scorecard revealed that only 13.3% of indicators are fit for use without caveats. Critically, 40.8% of indicators were duplicated across subcities, challenging the assumption of genuine subcity disaggregation. The health sector demonstrated the strongest subcity data collection capacity (38.9% duplication), whereas women’s affairs (83.3%), governance (80.0%), and social protection (66.7%) showed the highest duplication rates. Statistical validation using nonparametric tests confirmed significant differences across performance categories. Monte Carlo simulation validated nonparametric tests for small-sample (*n* = 4) data. On the basis of these findings, the following actions are recommended to improve the Seqota Declaration monitoring system and prepare for the 2026–2030 scale-up phase. First, the MoH and Addis Ababa City Administration should conduct a comprehensive data-quality audit before any performance assessment, using the framework developed in this study to identify which indicators are fit for use and which require redesign. Second, an investigation into the duplication across subcities should be prioritized within 3 mo, with particular attention to the women’s affairs (83.3% duplication), governance (80.0%), and social protection (66.7%) sectors, which showed the highest duplication rates and lack independent subcity data collection capacity. Third, indicators with degenerate variance (CV = 0%) should be redesigned or replaced within 6 mo, ensuring they capture meaningful variation over time that can support trend analysis and forecasting. Fourth, a complete baseline should be established for all indicators before the scale-up phase begins, as the systematic missingness of 2014 data currently prevents true baseline measurement for many indicators. Fifth, data-quality training should be provided for subcity offices within 6 mo, especially targeting sectors with high duplication rates, to build capacity for genuine subcity data collection and reporting. The replicable audit framework developed here can be applied in other settings adopting multisectoral nutrition monitoring systems, enabling evidence-based improvement of data quality before performance assessment.

## Author contributions

The authors’ responsibilities were as follows – GK, TS, DT, KA, YG: conducted research; GK, BA, ZS, AM, KA: analyzed data or performed statistical analysis; GK: primarily responsible for final content; and all authors: designed research, wrote the paper, provided essential materials and institutional support through their respective organizations, and read and approved the final manuscript.

## Data availability

Data described in the manuscript, code book, and analytic code will be made available upon request, pending application and approval from the Ministry of Health, Ethiopia.

## Declaration of Generative AI and AI-assisted technologies in the writing process

During the preparation of this work, the authors used Deep Seek in order to improve language refinement, grammar correction, and structural formatting for the body and tables. After using this tool/service, the authors reviewed and edited the content as needed and take full responsibility for the content of the publication.

## Funding

This research received no specific grant from any funding agency in the public, commercial, or not-for-profit sectors.

## Conflict of interest

All authors declare no conflicts of interest.
